# Biomass-derived molecules modulate the behavior of *Streptomyces coelicolor* for antibiotic production

**DOI:** 10.1007/s13205-016-0539-y

**Published:** 2016-10-15

**Authors:** Shashi Kant Bhatia, Bo-Rahm Lee, Ganesan Sathiyanarayanan, Hun Seok Song, Junyoung Kim, Jong-Min Jeon, Jeong-Jun Yoon, Jungoh Ahn, Kyungmoon Park, Yung-Hun Yang

**Affiliations:** 1 Department of Microbial Engineering, College of Engineering, Konkuk University, Seoul, 143-701 South Korea; 2 Institute for Ubiquitous Information Technology and Applications Konkuk University, Seoul, 143-701 South Korea; 3 IT Convergence Materials R&BD Group, Chungcheong Regional Division, Korea Institute of Industrial Technology (KITECH), 35-3 Hongchon-ri, Ipjang-myun, Seobuk-gu, Chonan-si, Chungnam 330-825 South Korea; 4 Biotechnology Process Engineering Center, Korea Research Institute Bioscience Biotechnology (KRIBB), Gwahangno, Yuseong-Gu, Daejeon, 305-806 South Korea; 5 Department of Biological and Chemical Engineering, Hongik University, Sejong Ro 2639, Jochiwon, Sejong, South Korea

**Keywords:** Antibiotic, Biomass, Furfural, *Streptomyces coelicolor*, Vanillin

## Abstract

Various chemicals, i.e., furfural, vanillin, 4-hydroxybenzaldehyde and acetate produced during the pretreatment of biomass affect microbial fermentation. In this study, effect of vanillin, 4-hydroxybenzaldehyde and acetate on antibiotic production in *Streptomyces coelicolor* is investigated. *IC*_50_ value of vanillin, 4-hydroxybenzaldehyde and acetate was recorded as 5, 11.3 and 115 mM, respectively. Vanillin was found as a very effective molecule, and it completely abolished antibiotic (undecylprodigiosin and actinorhodin) production at 1 mM concentration, while 4-hydroxybenzaldehyde and acetate have little effect. Microscopic analysis with field emission scanning electron microscopy (FESEM) showed that addition of vanillin inhibits mycelia formation and increases differentiation of *S. coelicolor* cells. Vanillin increases expression of genes responsible for sporulation (*ssgA*) and decreases expression of antibiotic transcriptional regulator (*redD* and *actII*-*orf4*), while it has no effect on genes related to the mycelia formation (*bldA* and *bldN*) and quorum sensing (*scbA* and *scbR*). Vanillin does not affect the glycolysis process, but may affect acetate and pyruvate accumulation which leads to increase in fatty acid accumulation. The production of antibiotics using biomass hydrolysates can be quite complex due to the presence of exogenous chemicals such as furfural and vanillin, and needs further detailed study.

**Electronic supplementary material:**

The online version of this article (doi:10.1007/s13205-016-0539-y) contains supplementary material, which is available to authorized users.

## Introduction

Lignocellulose is a complex-structured biomass composed of cellulose, hemicelluloses, and lignin. It cannot be used as carbon source directly and it should be available as fermentable sugar (Bhatia et al. 2015a, 2016; Verma et al. 2016). Various pretreatment methods using acids (such as sulfuric acid and phosphoric acid) or bases (such as ammonium hydroxide, sodium hydroxide, and potassium hydroxide) have been reported to improve the accessibility of enzymes to the polysaccharides embedded inside the cell wall and to release free sugars (Laurens et al. 2015; Patel et al. 2012; Verma et al. 2013). Lignocellulose pretreatment using different methods also lead to the release of different inhibitors, i.e., furfural, vanillin, 4-hydroxybenzaldehyde, and acetate, which further affects the ability of microbes to utilize free sugar as a carbon source (Allen et al. 2010). Furfural and vanillin are more toxic compounds beyond certain concentration and affect microbial growth (Zhang et al. 2014). A number of approaches have been used to investigate the mechanism of furfural action as this inhibits growth by damaging DNA and chemically reacting with cellular constituents (Akillioglu et al. 2011). Vanillin is one of the most prevalent phenolic compounds found in various lignocellulosic hydrolysates, e.g., from spruce, pine, poplar, corn stover and sugarcane bagasse, and acts as a quorum sensing (QS) blocker (Lv et al. 2014).

*Streptomyces coelicolor* is a commercially important actinomycete having the potential to produce two chemically distinct pigments as secondary metabolite, i.e., actinorhodin (Act, diffusible blue pigment) and undecylprodigiosin (Red), a cell wall-associated red pigment (Bhatia et al. 2016; Gomez-Escribano and Bibb 2014). The production of antibiotics is regulated by nutrients, growth rate, quorum sensing, transcriptional regulators and other pleiotropic genes (Liu et al. 2013). Regulation is influenced by various low molecular mass compounds, transfer RNA, sigma factors and gene products formed during post-exponential development. These events generate signals which affect a cascade of regulatory events resulting in chemical differentiation (secondary metabolism) and morphological differentiation (morphogenesis). The transcriptional regulation of each antibiotic’s biosynthetic gene cluster depends on a cluster-linked, antibiotic-specific and transcriptional regulator genes. Extensive classical and molecular genetic studies have led to the identification and characterization of numerous developmental genes, the *bld* and *whi*, and antibiotic-specific regulators, *actII*-*orf4* for Act and *redD* for Red (Bush et al. 2013; Lee et al. 2012; Price et al. 1999). Various genes responsible for the physiological controls which operate on pigment production in *S. coelicolor* are unknown. Molecular biology of antibiotic production is still not understood to a great degree and thus provides an opportunity for further investigation. Most of the research groups have reported that biomass-derived molecules act as an inhibitor, but interestingly in our previous research we found that furfural can elicit antibiotic production in *S. coelicolor* and it can be used to increase undecylprodigiosin production (Bhatia et al. 2016). Without considering various chemicals exist in biomass hydrolyzate, it is quite risky to use this as a carbon source. In this work, effect of other inhibitory molecules (vanillin, 4-hydroxybenzaldehyde and acetate) on antibiotic production and expression of various regulatory genes were studied.

## Materials and methods

### Chemicals

All the chemicals for media were purchased from Difco laboratories (Becton–Dickinson, Franklin Lakes, NJ, USA) and other chemicals, e.g., vanillin, 4-hydroxybenzaldehyde and acetate, were from Sigma-Aldrich (St. Louis, MO, USA). Agarose and bacterial agar were supplied by the Microbial carbohydrate resource bank at Konkuk University, Korea.

### Microorganism and seed culture

*Streptomyces coelicolor* A3 (2) M145 used in this study for secondary metabolite production was purchased from the Korean Culture Type Collection (KCTC), South Korea. *Streptomyces coelicolor* spores were cultivated on R5 agar plates for 72 h, harvested by scraping and suspended in 20 % (v/v) glycerol and stored at −80 °C (Kieser 2000). *Streptomyces coelicolor* seed culture was prepared by inoculating spores in 50 mL of LB liquid medium, with five glass beads of 3 mm size, and incubated at 30 °C under shaking condition (200 rpm). The germinated spores were harvested by centrifugation (3200×*g*, 4 °C, 10 min) and resuspended in 5 mL of ion-free water. 0.1 mL (2 × 10^6^ CFU) of germinated seed culture was used as inocula for further experiments in M9 minimal media of Difco laboratories with 1 % glucose as carbon source.

### Antibiotic extraction and quantification

For estimation of undecylprodigiosin (red) and actinorhodin (blue) antibiotics, 2 mL of culture samples was taken and divided into two aliquots. Actinorhodin estimation was performed by adding an equal volume of 1 M NaOH into one of the aliquot, centrifuged for 5 min at 4000*g*, and absorbance was taken at 633 nm. Undecylprodigiosin is a membrane-associated red pigment, so culture was harvested by centrifugation (4000*g* for 5 min) and cell pellet was suspended in methanol and incubated at 37 °C in a shaking incubator (200 rpm) for 1 h. Cells were removed by centrifugation at 4000*g* for 5 min; then 0.1 M HCl was added to the supernatant to adjust its pH and absorbance was measured at 533 nm. The concentration of actinorhodin and undecylprodigiosin was calculated as described already (Horinouchi and Beppu 1984).

### Inhibitors effect on *S. coelicolor* antibiotic production

In this study, the effect of biomass-derived inhibitors, vanillin, 4-hydroxybenzaldehyde and acetate, was investigated on growth and secondary metabolite production in *S. coelicolor*. To check the effect of various inhibitors, *S. coelicolor* was cultured in M9 minimal media of Difco laboratories, with 1 % glucose as carbon source and different concentrations of vanillin (0–1 mM), 4-hydroxybenzaldehyde (0–8 mM) and acetate (0–80 mM), for 72 h at 30 °C under shaking condition (200 rpm) at 10-mL scale. After 72 h, 2 mL of the culture sample was taken and biomass and antibiotics, i.e., undecylprodigiosin (Red) and actinorhodin (Blue), were estimated as mentioned above.

### Field emission scanning electron microscopy (FESEM)

Vanillin was observed as the most effective molecule which drastically changes the antibiotic production in *S. coelicolor* at very low concentration. To study the effect of this compound further, *S. coelicolor* was cultured using minimum effective concentration of vanillin (1 mM) as mentioned above and monitored for morphological change. Samples were prepared for FESEM analysis using methods as already reported (Ishii et al. 2004). FESEM was performed by SUPRA 55VP, CarlZeiss, Oberkochen, Germany. The samples were monitored with a 15-kV accelerating voltage and photographic images were captured digitally at different magnification.

### Lipids and metabolite quantification

Antibiotic and fatty acid production pathways are interrelated (Revill et al. 1996), so total fatty acid of *S. coelicolor* cultured with and without vanillin was extracted and analyzed for composition as described already (Bhatia et al. 2015b). For metabolite analysis, *S. coelicolor* was cultured with vanillin at 30 °C for 72 h. On completion of growth 1 mL of sample was collected, centrifuged at 12,000*g* and supernatant was analyzed using an HPLC system equipped with a Bio-Rad Aminex HPX-87H column (Bio-Rad Co., Hercules, CA, USA). A mobile phase of 5 mM H_2_SO_4_ at a flow rate of 0.6 mL/min was used and the column temperature was maintained at 50 °C. Various organic acids were quantified at 210 nm.

### Antibiotic regulatory genes study

There are various genes of *S. coelicolor* already reported having a role in quorum sensing, secondary metabolite and morphology development, which altogether affects antibiotic production (Table 1). To study the mRNA expression level of these genes in the presence of vanillin, RT-PCR analysis was performed. For mRNA extraction, *S. coelicolor* spores were germinated for 5 h in LB broth at 30 °C and further used as seed. *Streptomyces coelicolor* culture was grown in the presence of vanillin (1.0 mM) at 10-mL scale for 72 h at 30 °C and samples were withdrawn at different time intervals. Collected samples were rapidly cooled on ice in pre-chilled Falcon tubes after which they were centrifuged at 4000*g* for 10 min at 4 °C. The supernatant was discarded and the pellet was added with 0.5 mL of RNA protect reagent (Qiagen, Valencia, CA, USA). The mixture was then incubated at 25 °C for 5 min. The RNeasy mini kit (Qiagen) was used for extracting total RNA from the control and vanillin-affected samples. The RNA was quantified using NanoDrop (Thermo Fisher Scientific, Waltham, MA, USA) by measuring the absorbance at 260 and 280 nm. Super-script II reverse transcriptase kit (Invitrogen, Carlsbad, CA, USA) was used for cDNA synthesis from the extracted RNA.

**Table 1:** Various genes targeted for the mRNA expression and primers designed for their amplification to study the effect of vanillin on their expression level

Gene ID	Gene	Primer
Antibiotic synthesis genes		
SCO5877	*redD*	RT_redD_F: CCCGACAACGTCCTCAAC RT_redD_R: CGAGACGAGTCTCAGGAAGC
SCO5085	*actII*-*orf*4	RT_actII4_F: AGAATAGGGCCGATGATTCC RT_actII4_R: CCCAGTTCGTCGGACAGTAT
Morphological genes		
SCOt24	*bldA*	RT_bldA_F: GCCCGGATGGTGGAATGCAG RT_bldA_R: TGGTGCCCGGAGCCGGACTT
SCO3323	*bldN*	RT_bldN_F: CCTCGAGTCCCTCTCCAAC RT_bldN_R: CGGTACTGGAGCGTTTTGAT
SCO3926	*ssgA*	RT_ssgA_F: CCTTTCATCTGCCCGGAGAC RT_ssgA_R: CGACCTGAAGTCGGATCAGC
SCO1541	*ssgB*	RT_ssgB_F: TCGTGTGCATCGCTCTCAG RT_ssgB_R: CTAGCTTTCCGCCAGGATGT
SCO3925	*ssgR*	RT_ssgR_F: GGCTGTTCTTCCTCGGTGAG RT_ssgR_R: GAGACGCACATGACCTCGAT
SCO2082	*fstz*	RT_ftsZ_F: GTTCATCGCCATCAACACCG RT_ftsZ_R: TGTCACGAAGACCATGTCGG
Quorum sensing and pleiotropic genes		
SCO6266	*scbA*	RT_scbA_F: ACTACACCTGCCACCTCGAC RT_scbA_R: GCCGGTAGACTTGAGGACTG
SCO6265	*scbR*	RT_scbR_F: TCTTCGAGAAGCAGGGCTAC RT_scbR_R: GCCCATGTCGATGAGTTCTT
SCO4425	*afsS*	RT_afsS_F: ATGAGCGACAAGATGAAGGA RT_afsS_R: GGTTGTCCATCGTGGTGAT
SCO4426	*afsR*	RT_afsR_F: GGCTGCTGGACTTCTACCTG RT_afsR_R: CCTCCGTGTACAGCCAGTC

### Reverse transcription polymerase chain reaction (RT-PCR)

The primers specific for various genes and for the endogenous control (16S rRNA) were designed using the Primer Express software^®^ (Applied Biosystems, Foster City, CA, USA) based on *S. coelicolor* genome published in the NCBI database. Custom-synthesized primers for each gene were obtained from Integrated DNA Technologies (Foster City, CA, USA). The primers used in the study are provided in Table 1. RT-PCR was done with the LifePro thermal cycler by custom thermal cycling conditions with the normalized cDNA as a template. The samples were analyzed in the duplicates and standardized against 16S rRNA gene expression. The relative changes in mRNA expression levels were determined using comparative band density between the vanillin and control *S. coelicolor*.

## Results and discussion

### Effect of inhibitors on antibiotic production and morphology

**Fig. 1:**
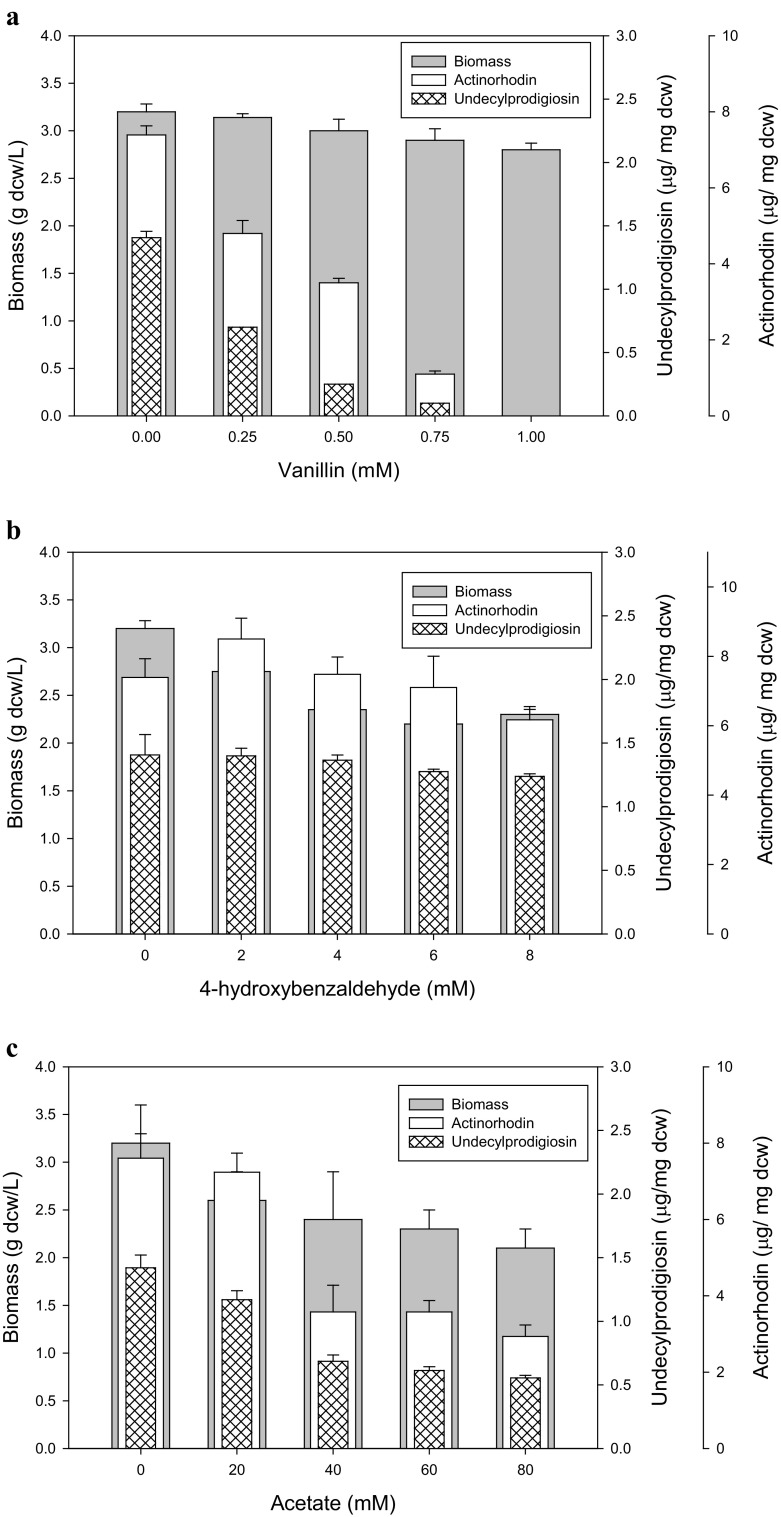
Effect of biomass-derived chemicals **a** vanillin, **b** 4-hydroxybenzaldehyde (4-HB) and **c** acetate on *S. coelicolor* growth and antibiotic production. *S. coelicolor* was cultured in M9 media with 1 % glucose and various concentrations of inhibitors

Furfural, vanillin, 4-hydroxybenzaldehyde and acetate are chemical molecules produced during the pretreatment of biomass (Allen et al. 2010). To know the effect of vanillin, 4-hydroxybenzaldehyde and acetate on antibiotic production in *S. coelicolor*, different concentrations were investigated. Vanillin had little effect on biomass production while a rapid decrease in antibiotic (undecylprodigiosin and actinorhodin) production was recorded with the increase of its concentration. There was no antibiotic production observed above 0.75 mM vanillin (Fig. 1a). 4-Hydroxybenzaldehyde and acetate have a mild effect on biomass and antibiotic production in *S. coelicolor* (Fig. 1b, c). *IC*_50_ value for vanillin, 4-hydroxybenzaldehyde and acetate was calculated as 5.0, 11.3 and 115 mM, respectively. *Streptomyces coelicolor* cells were cultured in the presence of various inhibitors at their *IC*_50_ concentration and antibiotics (undecylprodigiosin and actinorhodin) were extracted. In the presence of vanillin, no antibiotic was observed; however, in case of 4-hydroxybenzaldehyde and acetate, reduction in both antibiotics’ production was observed as compared to control (Fig. S1). Other biomass-derived molecule such as furfural enhanced undecylprodigiosin production and inhibits actinorhodin production in *S. coelicolor* as already reported (Bhatia et al. 2016). In this study, vanillin was found as a most effective molecule which affected antibiotic production in *S. coelicolor* dramatically (Fig. S1). It was not easy to expect why this phenomena happened because there is no report on vanillin effect.

**Fig. 2:**
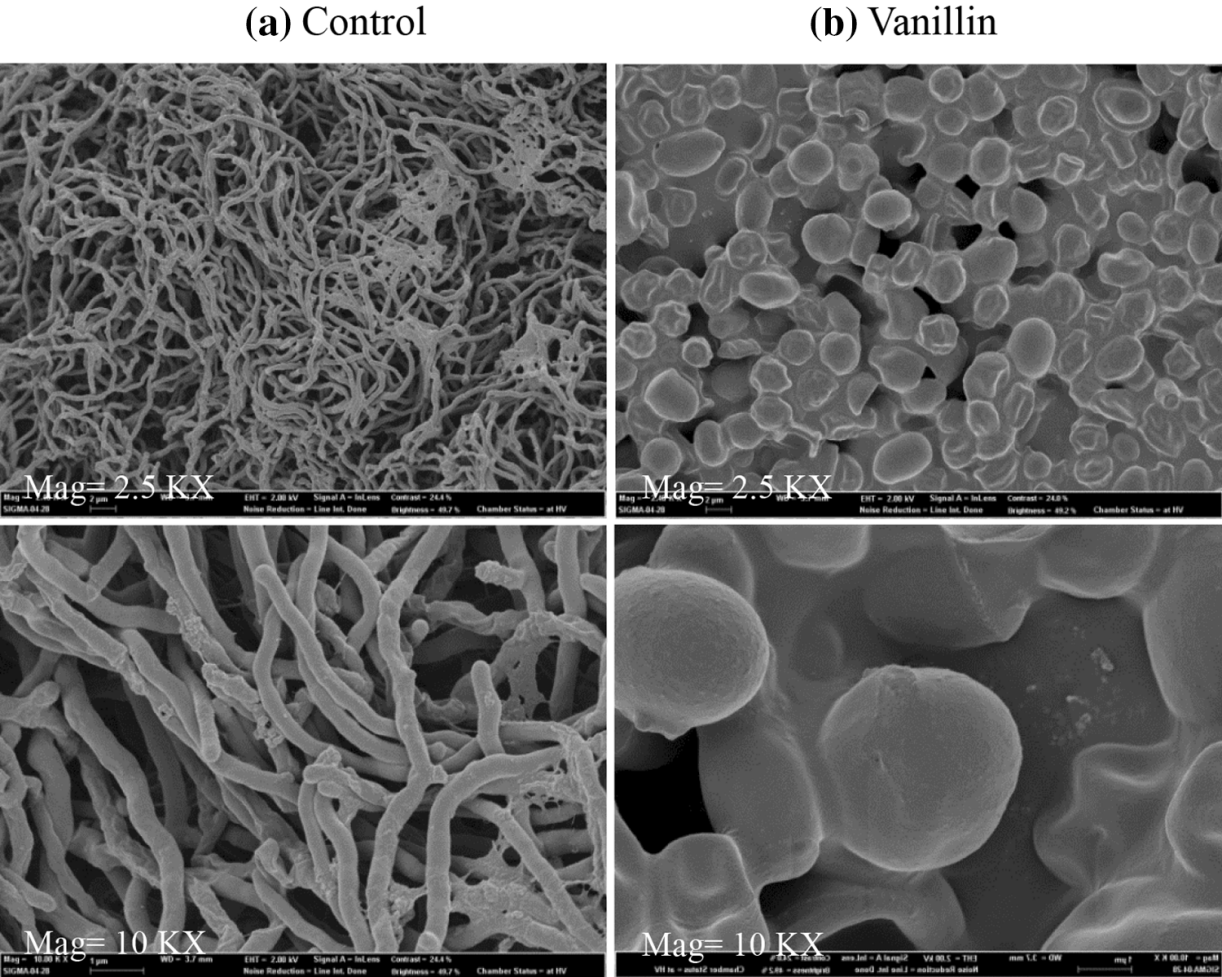
Field emission scanning electron microscopy (FESEM) of *S. coelicolor*. **a** Control: normal cell morphology with mycelia; **b** with vanillin: cells are *round shaped* and mycelia formation is inhibited

Among examined inhibitors, as vanillin showed inhibitory effects on antibiotic production at low concentration, any morphological change was further investigated. Field emission scanning electron microscopy (FESEM) of *S. coelicolor* cells cultured in the presence of vanillin was performed. *Streptomyces coelicolor* cells without any inhibitors showed normal mycelial growth and morphology (Fig. 2a). *Streptomyces coelicolor* cells grown in the presence of vanillin showed inhibition of mycelia formation (Fig. 2b), and cells had a round structure. In the presence of vanillin, *S. coelicolor* cells are unable to produce antibiotics due to the lack of mycelia. Mycelia formation is required for polyketide antibiotic production in *S. coelicolor* as already reported (Gehring et al. 2001).

### Transcriptional analysis of antibiotic regulatory gene

*Streptomyces coelicolor* cultured in the presence of vanillin completely abolished antibiotic production. mRNA expression profile of *redD* and *actII*-*orf4* was analyzed at 48- and 72-h intervals and reduction in expression level was observed (Fig. S2). *redD* and *actII*-*orf4* are transcriptional activator genes of the undecylprodigiosin and actinorhodin biosynthetic pathway, respectively, and regulate antibiotic production (Fujii et al. 1996; Wang et al. 2014). Bhatia et al. reported that increase in undecylprodigiosin and reduction in actinorhodin production in *S. coelicolor* under the effect of furfural is due to altered expression of *redD* and *actII*-*orf4* (Bhatia et al. 2016). There was no change in the mRNA expression level of genes responsible for mycelia formation (*bldA, bldN*) analyzed in the presence of vanillin during all growth phases (Fig. S2). *BldA* has a role in mycelia development in *S. coelicolor* and it codes tRNA for leucine codon UUA required for undecylprodigiosin production at higher phosphate concentrations (White and Bibb 1997). B*ldN* gene codes for sigma factor (BldN) required for the formation of specialized spore-bearing aerial hyphae during differentiation in the mycelial bacterium *S.* *coelicolor* (Bibb and Buttner 2003). Genes responsible for sporulation and differentiation of *S. coelicolor* were investigated for mRNA expression level. An increase in the *ssgA* mRNA expression level (1.6-fold) was recorded. The *ssgA* gene involved in cell division and development as already reported (van Wezel et al. 2000). Other genes responsible for sporulation, i.e., *ssgB*, *ssgR* and *ftsZ*, were not affected by vanillin (Fig. S2). Various genes involved in quorum sensing which may affect antibiotic production were also studied. Vanillin had no effect on *scbA* and *scbR* expression levels (Fig. S2). Change in *scbA* expression has no role in secondary metabolite production as already reported (D’Alia et al. 2011); an *scbA* mutant that failed to produce gamma-butyrolactones can still produce antibiotics, i.e., actinorhodin (Act) and undecylprodigiosin (Red). The decrease in *scbR* expression leads to a delay in undecylprodigiosin antibiotic production, as *scbR* failed to make gamma-butyrolactones (D’Alia et al. 2011). *AfsS* and *afsR* are pleiotropic genes that regulate undecylprodigiosin and actinorhodin synthesis pathways (Horinouchi 2003; Lian et al. 2008). The increase in *afsR* copy number can stimulate both Act and Red production (Floriano and Bibb 1996), but no changes in the mRNA expression level of *afsS* and *afsR* were recorded in the presence of vanillin. From mRNA expression data of morphological genes, it could be concluded that vanillin is affecting genes involved in sporulation and enhances differentiation of cells shown by FESEM analysis result.

### Total lipid and metabolite profiling

*Streptomyces coelicolor* cells were cultured with and without inhibitors in the above-mentioned conditions. *Streptomyces coelicolor* without any inhibitors was able to accumulate fatty acid (6.3 µg/mg dcw), while vanillin addition resulted in an increase in fatty acid accumulation (19 µg/mg dcw) (Table 2). Vanillin inhibited antibiotic production and resulted in an increase in fatty acid accumulation in *S. coelicolor*, as antibiotic and fatty acid synthesis pathways are interrelated (Revill et al. 1996). Metabolite concentrations in *S. coelicolor* culture supernatant were quantified at 72 h. Glucose was consumed almost completely in control while 6 % reduction in glucose utilization was recorded in the presence of vanillin. Increase in acetate (2.6 mM) and pyruvate (4.7 mM) accumulation was observed in the presence of vanillin (Fig. S3). This observation suggests that glycolysis pathway of *S. coelicolor* in control and vanillin-treated cell is working properly as there was little change in glucose consumption. Vanillin represses the expression of antibiotic synthesis genes and enhances acetate accumulation which further reduces antibiotic production as observed above with the external addition of acetate. Organic acid content, i.e., acetate and pyruvate, affects the pH of fermentation broth which further changed antibiotic synthesis in *S. coelicolor* as already explained (Yang et al. 2010).

**Table 2:** Total fatty acid profile of *S. coelicolor* under the effect of vanillin

Fatty acid	Control (%)	Vanillin (%)
C12:0-3OH	3.3 ± 0.1	4.6 ± 0.4
C14:0-13M	22.0 ± 2.3	18.3 ± 3.4
C15:1	2.0 ± 0.2	1.76 ± 0.05
C14:0-2OH	0.62 ± 0.04	0.49 ± 0.02
C14:0-3OH	29.2 ± 4.0	33.54 ± 2.9
C15:0-14M	3.0 ± 0.07	2.26 ± 0.7
C16:1-n9	19.0 ± 3.3	22.6 ± 1.3
C16:0 cyclo	18.7 ± 2.7	14.30 ± 2.1
C16:0-15M	1.30 ± 0.06	1.0 ± 0.06
C16:0-2OH	0.45 ± 0.02	0.28 ± 0.02
C18:3-n6,9,12	0.21 ± 0.01	0.58 ± 0.04
C18:1-n9t	0.36 ± 0.05	0.27 ± 0.01
Total fatty acid (µg/mg dcw)	6.2 ± 0.8	19 ± 2.3

## Conclusion

Biomass is an abundantly available raw material and can be used to develop an economic bioprocess for the production of industrial valuable compounds. Use of biomass hydrolysate without knowing its composition may lead to adverse effects on microbial fermentation and productivity. Cultivation of *S. coelicolor* in the presence of biomass-derived molecules affects metabolite pool and morphology of *S. coelicolor,* which further leads to change in antibiotic production. Microbes show different behavior against each inhibitory compound; therefore, there is a need to study the role of such type of compounds to use biomass as a potential carbon source.

## Electronic supplementary material

Below is the link to the electronic supplementary material.

Supplementary material 1 (DOCX 244 kb)
