# Supplementary material for: Biomass-derived molecules modulate the behavior of Streptomyces coelicolor for antibiotic production
Source: 3 Biotech. 2016 Oct 15;6(2):223. doi: 10.1007/s13205-016-0539-y (PMC5065882; doi:10.1007/s13205-016-0539-y)
Supplement: Supplementary file 1 — Supplementary material 1 (DOCX 244 kb) [file 13205_2016_539_MOESM1_ESM.docx]

**Fig. S1** Picture shows the change in antibiotics production (undecylprodigiosin (red) and actinorhodin (blue)) under the effect of various inhibitors at their respective IC_50_ value, i.e., vanillin (5 mM), 4-HB (11.3 mM) and acetate (115 mM) in M9 media with glucose (1%) as the main carbon source. Upper figure shows antibiotic production in the culture broth and the lower figure represents extracted antibiotics e.g. undecylprodigiosin and actinorhodin.

**
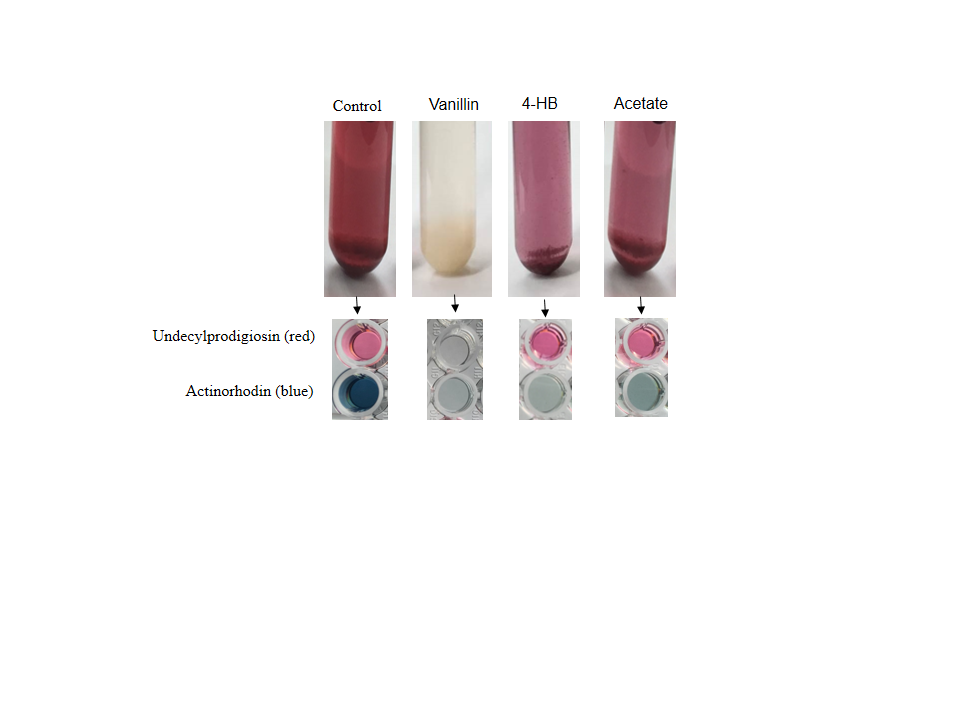
**

**Fig. S2** Effect of vanillin on the mRNA expression of various genes involved in antibiotic synthesis at 48 h and 72 h time interval.

**Fig. S3** Residual glucose and metabolite analysis of *S. coelicolor* under the effect of vanillin at 72 h. *S. coelicolor* was cultured in M9 media with 1% glucose and 1 mM vanillin at 30 °C. The bar represents glucose, is the residual concentration after completion of fermentation. Other bars represent the concentration of various organic acid accumulated during the fermentation.
